# Network structure of scientific collaborations between China and the EU member states

**DOI:** 10.1007/s11192-017-2488-6

**Published:** 2017-08-21

**Authors:** Lili Wang, Xianwen Wang, Niels J. Philipsen

**Affiliations:** 1 UNU-MERIT, Maastricht University, Boschstraat 24, 6211 AX Maastricht, The Netherlands; 2 WISE Lab, Dalian University of Technology, Dalian, 116024, People’s Republic of China; 3 METRO, Maastricht University, 6200 MD Maastricht, The Netherlands; 4 RILE, Erasmus University Rotterdam, 3000 DR Rotterdam, The Netherlands

**Keywords:** International collaboration, Science, Network, Structure, Centrality, Integration, EU member states

## Abstract

Collaborations between China and the European Union (EU) member states involve not only connections between China and individual countries, but also interactions between the different EU member states, the latter of which is due also to the influence exerted by the EU’s integration strategy. The complex linkages between China and the EU28, as well as among the 28 EU member states, are of great importance for studying knowledge flows. Using co-authorship analysis, this study explores the changes of the network structure between 2000 and 2014. Our results show that EU member states with middle- or low- scientific capacities, in particular those who joined the EU after 2000, have been actively reshaping the network of scientific collaborations with China. The linkages between middle- and low- scientific capacity countries have been tremendously strengthened in the later years. The network positional advantage (measured by the degree of betweenness centrality) has shifted from a few dominant nations to a wider range of countries. We also find that countries like Belgium, Sweden and Denmark are in important positions connecting the relatively low-capacity ‘new’ EU member states with China. The ‘new’ EU member states—that have relatively low scientific capacity—intend to cooperate with China jointly with ‘old’ EU member(s).

## Introduction

Committed to promoting science and technology and increasing China’s global influence, the Chinese government has placed great emphasis on international cooperation. In recent decades, China has gradually established science and technology collaborations with more than 150 countries and signed cooperation agreements with nearly 90 countries (Zhou and Glänzel 2010). In evaluating collaboration results, however, the existing literature has been mainly focusing on collaborations between China and the U.S (Wang et al. 2012, 2013; Tang 2013; Tang and Shapira 2011; Suttmeier 2014; Hand 2010). Little is known about collaborations between China and its second biggest partner, the European Union. In recent years, along with the fast and comprehensive growth of China-EU relations, the collaboration in S&T between China and European countries has also grown rapidly, which could be reflected by increased mobility of people (including scientists), collaboration in research projects, and coauthorship in publications (Li and Chang 2014). Neverthess, the limited research on scientific collaborations between China and the EU so far has been mainly conducted at the national level, for instance in relation to the UK (Bound et al. 2013; Zhou et al. 2013).

In fact, the European countries have a very high national share of international collaborations (Zhou and Glänzel 2010; Hand 2010). The EU level of international co-authorship is about twice that of the United States (Hand 2010). Nevertheless, due to the funding programmes from the European Commission and the spatial proximity in Europe, the pattern of intra-EU collaboration is different from that of extra-EU collaboration (Mattsson et al. 2008; Tijssen 2008). In the process of collaborating with China, the interaction between the heterogeneous 28 member states of the European Union (EU28) is of great interest.

The contribution of this study is twofold. First, from the *extra*-*EU* perspective, this study aims to provide an in-depth understanding on the evolution of collaboration networks between China and the 28 EU member states. Mapping the change of the network structure is crucial in advancing future knowledge transfers between China and the EU28. Second, from the *intra*-*EU* perspective, this study offers insights on the interactions between European countries. By analyzing the network structure of scientific collaborations, we investigate whether European countries have been (further) integrating while collaborating with China. If so, to what extent (and in what scope) such integration is happening? Special attention will be paid to the change of collaboration patterns of ‘new’ member states,^1^ as well as the interactions between the ‘new’ and ‘old’ member states.

The remainder of this paper is organized as follows. In “Backgrounds” section summarizes the background of China-EU cooperation and reviews related literature. In “Data and Methodology” section  provides data and methodology. Analyses and results are documented in “Results” section and “Conclusions” section concludes.

## Backgrounds

### Cooperation efforts from China and the EU

Along with its rapid economic growth and social change, China has been seeking to integrate into international structures (European Commission 1995). Aiming at more bilateral cooperation, China and the EU signed the Science and Technology Agreement in December 1998, which was renewed in 2004, 2009 and 2014.^2^ Jointly steered by DG Research and Innovation and the Chinese Ministry of Science and Technology (MoST), the European Atomic Energy Community (EURATOM) and China signed an agreement for R&D Cooperation in the Peaceful Uses of Nuclear Energy (R&D-PUNE Agreement) in April 2008.^3^ In order to complement and strengthen further fruitful scientific cooperation, MoST and DG Research and Innovation signed the Agreement on Implementing the Science and Technology Partnership Scheme (CESTYS) in May 2009.^4^

As the largest research funding agency for basic research and application-oriented research in China, NSFC (National Natural Science Foundation of China) signed the Agreement of Scientific and Technological Cooperation between Chinese government and the European Community in 1998, which was renewed in 2009, aiming to launch projects in specific research areas of common interest. Many other bilateral agreements between NSFC and the EU countries or various funding agencies in the EU have been reached, e.g. agreements with DFG (German Research Foundation), ANR (French National Agency for Research), FWO (Research Foundation—Flanders), STINT (Swedish Foundation for International Cooperation in Research and Higher Education), FWF (Austrian Science Fund), NWO (Netherlands Organization for Scientific Research), etc.

The European Union has also made efforts in promoting the cooperation with China. The EU’s Framework Programmes, including the FP6 (6th Framework Programme for Research and Technological Development, 2002–2006), FP7 (2007–2013) and Horizon 2020 (2014–2020) programmes are all fully open to international cooperation, and China is the most important cooperation country in these programmes (European Commission 2016).

Being aware of the fact that China was in the midst of sustained and dramatic economic change and that China has been seeking integration into international structures, the European Commission (1995) issued an official document—A long term policy for China–Europe relationship—listing China’s importance for Europe and stressing that “Europe must develop a long-term relationship with China that reflects China’s worldwide, as well as regional, economic and political influence”.

Owing to the efforts and cooperation agreements from both sides, scientific collaborations between China and the European Union have been growing dramatically. The number of papers co-authored by Chinese researchers and researchers affiliated in the EU28 has increased from 2500 in 2000 to more than 19,000 in 2014. For most EU member states, China is the second most extra-EU collaborating country only after the USA in 2016.^5^ Despite the rapid growth in collaborations, however, little is known about the structure of collaboration networks between China and the EU, nor the dynamics of how EU member states interact while collaborating with China.

### International scientific collaborations

Collaborations have been widely recognized as a more and more common phenomenon in science. Through collaboration, partners can share knowledge, skills, techniques and improve productivity (Katz and Martin 1997; Beaver and Rosen 1978; Price and Beaver 1966). International collaborations have been believed to generate even higher social impacts than domestic collaborations. Based on a set of papers published in 1995 and 1996, Glänzel (2001) points out that international co-authorship generally results in publications with higher citation rates than purely domestic papers. Using a database containing nearly a half million refereed UK publications, Katz and Hicks (1997) find that domestic collaborations (with authors from the same institution or another domestic institution) increase the average impact by approximately 0.75 citations while international collaborations (with authors from foreign institution) increase the impact by about 1.6 citations. With a set of SCI papers published by European institutes, Narin et al. (1991) find that the citation received by internationally co-authored papers is twice as high as that received by papers authored by scientists working at a single institution within a single country. Nomaler et al. (2013) stress the factor of geographical distance, pointing out that collaborations between geographically distant researchers tend to receive higher citations.

In China, the 15-year “Medium- and Long-Term Programme for Science and Technology Development” issued in 2006 demonstrated that one of the primary goals by 2020 is to have the average cited scientific publications of Chinese authors reach the top 5 worldwide.^6^ As citation improvement relies on earlier reputation and recognition (Wang 2016), this goal may be difficult to be fulfilled only by domestic publication. In this regard, publishing jointly with international scholars would be beneficial for Chinese scholars to advance their recognition in particular outside of China. By tracking corresponding authors of the joint publications between China and the EU28, Wang and Wang (2017) find that the academic cooperation between China and the EU has been mainly set up by Chinese researchers, and in the fast-growing collaborative fields the scores of revealed comparative advantages have all improved in China.

In the process of collaborating with China, the intra-European collaboration is also of great importance, which has been embodied by the European Union’s strategic goal to strengthen regional cohesion in the EU and stimulated work towards an European Research Area (ERA) (European Council 2000). In line with the aim of integrating and coordinating research activities at national and Union level, scientific communities of Western and Eastern Europe are in particular encouraged to be integrated (European Commission 2000). According to the ERA survey, the national research systems of the EU member states have become more aligned to the ERA priorities, and member states have been increasingly open to international cooperation (European Commission 2014). Based on scientific publications between 1998 and 2004, Hoekman et al. (2009) find that collaborations between European regions have been impeded by geographical barriers, and the research activities in the EU is far from being integrated. Tijssen (2008) shows that some EU member states have a relatively strong preference for research partners.

Given the great heterogeneity of European countries, country size and capability should be taken into consideration in studying collaborations with and within Europe. Lacking of collaborative partners within the national borders, smaller European countries are expected to collaborate more internationally (Frenken 2002). With a development model, Moed (2016) argues that the share of a country’s internationally co-authored articles is dependent on the phase of a country’s scientific development. Internationally co-authored publications are expected to grow very fast in a country’s building-up stage, which could explain the fast growth of the international collaboration of China during the last two decades.

In measuring research collaborations, several types of indicators are often used. First, bibliometric analysis of co-publications is regarded as “one promising approach” (Melin and Persson 1996) and it “provides a window on patterns of collaboration” (Newman 2004). This approach has been widely applied in examining collaborations at country level (Beaver and Rosen 1978; Katz and Hicks 1997; Glänzel 2001; Coccia and Wang 2016; Wang and Wang 2017; Wang et al. 2013), at regional/city level (Hoekman et al. 2009), and institution or author level (Yan and Guns 2014; Wang et al. 2013). Following that, co-inventorship is another often used as an indicator in analysing research collaboration (Guan et al. 2015; Morescalchi et al. 2015; Gao et al. 2011; Hoekman et al. 2009). Gao et al. (2011) state that co-publications and co-inventions are the main types of outcome of research collaboration. A third group measures research collaboration by the number of joint research and development (R&D) projects (Scherngell and Barber 2011; Scherngell and Lata 2013; Revilla et al. 2003; Autant-bernard et al. 2007; Hazir and Autant-Bernard 2014). Joint participations in projects reflect the common R&D activities between partners. It is worth noting that, as pointed out by Melin and Persson (1996), not all research collaboration necessarily leads to one type of output (e.g. co-authored papers, co-inventions or R&D projects). Among these indicators, co-authorship of articles has been widely accepted to measure collaboration within the academic community (Melin and Persson 1996; Newman 2004).

### Structure of collaboration networks

The structure of collaboration networks reveals the pattern of knowledge exchange and the mechanism of preferential attachment (Wagner and Leydesdorff 2005). In scientific collaboration networks, important changes have been predicted to be under way (Toivanen and Ponomariov 2011). Adams (2012) states that the new “regional networks are reinforcing the competence and capacity of emerging research economies, and changing the global balance of research activity” (Adams 2012, p. 335).

Strong ties have been believed to be more important for exchanging knowledge than weak ties (Fritsch and Kauffeld-Monz 2010). Lacking of collaboration tendency, scientific super powers may lose their advantageous position in the global networks (Adams 2012). Leydesdorff and Wagner (2008) find that the global collaboration network has reinforced the formation of a core group consisting of fourteen most cooperative countries during the period 2000–2005, and this core group is expected to use knowledge from the global collaboration networks with great efficiency.

Toward tracking knowledge flows, Breschi and Lissoni (2003) argue that connection in the social network (known as social proximity) is of more importance than closeness in geography (known as spatial proximity), which is confirmed by Autant-bernard et al. (2007): “social distance seems to matter more than geographical distance”. Hoekman et al. (2010) find that the ongoing process of European integration is removing territorial borders (regional, national, language) on the intensity of research collaboration across European regions. Scherngell and Lata (2013) also confirm that geographical distance and country border effects on the collaborations among 255 European regions gradually decrease over the period 1999–2006. Following this theory, despite the close distance between some European countries, the “active participation in a network” is the key for knowledge exchanges. To this end, exploring the China-EU network structure provides a deeper understanding on knowledge flows not only between China and the EU, but also between European regions.

## Data and methodology

There are various methods of measuring research collaborations, as discussed in “International scientific collaborations” section. In this study, we adopt the co-authorship measurement. Data are collected from Science Citation Index Expanded (SCI-E) and Social Sciences Citation Index (SSCI) of Thomson Reuters (currently known as Clarivate Analytics). In our analysis, we focus on the international collaborations at national level. Affiliation address is used to identify the location of authors.

In exploring international scientific collaborations, one needs to “go beyond absolute differences in country sizes and estimate *propensities* or *intensities* of collaboration” (Luukkonen et al. 1993). In this regard, Jaccard and Salton indexes are the two commonly used measures to capture the relative strength of bilateral connections between countries (Luukkonen et al. 1993; Leydesdorff 2008; Boschma et al. 2014). Another type of indicator, the Probabilistic Affinity Index (PAI), introduced by Zitt et al. (2000), calculates the ratio of observed and expected number of links. Similar to PAI, Probabilistic Partnership Index (PPI) also measures the deviation from expected values of collaborative linkages (Yamashita and Okubo 2006). The difference between PAI and PPI is that the former measures the expected value from the number of links while the latter is calculated from the current participants (see more details in Yamashita and Okubo 2006). Both PAI and PPI methods seem to be extremely sensitive to the level of breakdown of collaborating partners (Zitt et al. 2000; Zitt and Bassecoulard 2004; Yamashita and Okubo 2006). In our study examining collaborations between China and multiple European countries at the same time, we adopt the first set of measurements, i.e. Jaccard or Salton index. Luukkonen et al. (1993) states that “the Jaccard measure is preferable to Salton’s measure since the latter underestimates the collaboration of smaller countries with larger countries”. Leydesdorff (2008) also suggests that “Jaccard index is the best basis for the normalization because this measure does not take the distributions along the respective vectors into account”. Considering the size difference between China and most European countries, the Jaccard index is a more suitable choice for our study.

The collaboration strength index using Jaccard’s measure (Luukkonen et al. 1993) can be expressed as:

1 $$C_{ij} = \frac{{\text{CO}_{ij} }}{{P_{i} + P_{j} - \text{CO}_{ij} }}\quad \left( {i \ne j} \right)$$

where $\text{CO}_{ij}$ is the number of co-authored papers between country *i* and country *j*; $P_{i}$ is the number of total publication by country *i*; $P_{j}$ is the number of total publication by country *j*.

We have further compared the Jaccard index and the Salton index^7^ in our dataset. Applying both methods to our data set yields similar results. Given that regional difference is slightly more pronounced in the results produced by the Jaccard index, we choose this index in our analysis.

Following Eq. (1), calculating the bilateral connections between each pair of the 29 countries (i.e. China and 28 European countries) produces a 29 * 29 collaboration matrix. We repeat this calculation for different years to derive a set of dynamic matrices. To avoid exceptional cases in the network evolvement, we use a five-year window scheme. We use the general structure in 2000–2004 to represent the earlier stage, and the one in 2010–2014 to represent the later stage. Hence the Network evolution is captured by a comparison of network changes between the two periods, i.e. the period of five earlier years (2000–2004) and the period of five later years (2010–2014).

In order to examine the change of central players in the network, we calculate the betweenness centrality for both stages. The betweenness centrality of country *i* is defined as:

2 $$C_{t} \left( i \right) = \sum \frac{{P_{sk,t} \left( i \right)}}{{P_{sk,t} }}\left( {s = {\text{country}}1, {\text{country}}2, \ldots {\text{country}}29;\;k = {\text{country}}1, {\text{country}}2, \ldots {\text{country}}29;\;i \ne s \ne k } \right)$$

$P_{sk}$ is the number of shortest paths from country *s* to country *k* at year *t*. $P_{sk} \left( i \right)$ is the number of shortest paths from country *s* to country *k* that contain country *i* at year *t*.

In order to keep the degree of betweenness centrality comparable over time, we normalize the above centrality value to a scale within 0 and 1.

3 $$C_{t}^{n} \left( i \right) = \frac{{\hbox{max} \left( {C_{t} } \right) - C_{t} \left( i \right)}}{{\hbox{max} \left( {C_{t} } \right) - { \hbox{min} }\left( {C_{t} } \right)}}$$

where $C_{t}^{n} (i)$ is the normalized betweenness centrality of country *i* at year *t*; ${ \hbox{max} }\left( {C_{t} } \right)$ is the maximal value of betweenness centrality of all the 28 EU countries at year *t*; ${ \hbox{min} }\left( {C_{t} } \right)$ is the minimal value of betweenness centrality of all the 28 EU countries at year *t*.

## Results

### Dynamics of scientific collaborations

During the period between 2000 and 2014, there were in total over 123,800 joint publications between China and the EU 28, among which the top three collaborating European countries with China were the UK (42,561), Germany (33,352), and France (20,401), followed by the Netherlands (10,455), Italy (10,257) and Sweden (9860). These six countries jointly published with China 107,459 articles in total, accounting for nearly 87% of all China-EU28 joint publications.

It is noteworthy that the European Union has evolved over the years. Formed by six countries^8^ in 1958, the EU has gradually expanded to 28 member states by 2014. Thirteen countries joined the EU in the 2000s, namely Czech Republic, Estonia, Cyprus, Latvia, Lithuania, Hungary, Malta, Poland, Slovenia and Slovakia in 2004, Bulgaria and Romania in 2007, and Croatia in 2013.^9^ Due to the time difference in joining the EU, different collaboration patterns have been observed between the established 15 member states and the 13 new members (Makkonen and Mitze 2016). Following Makkonen and Mitze (2016), we disentangle the established 15 ‘old’ EU member states^10^ which joined the EU before 2004 from the 13 ‘new’ ones.

Given that country sizes and research capacity vary greatly among the European countries, propensities toward international collaborations are also different across nations. In terms of absolute co-publication counts, regions with large research outputs are believed to collaborate more (Hoekman et al. 2010). However, to capture the relative strength of links between countries, it is necessary to eliminate the country-size effect (Luukkonen et al. 1993). As explained in “Data and methodology” section, the propensity of collaboration in this study is measured by intensity instead of absolute publication numbers. Before the network analysis, we first examine the propensity of international collaborations, with a connection of scientific capacity.

**Fig. 1:**
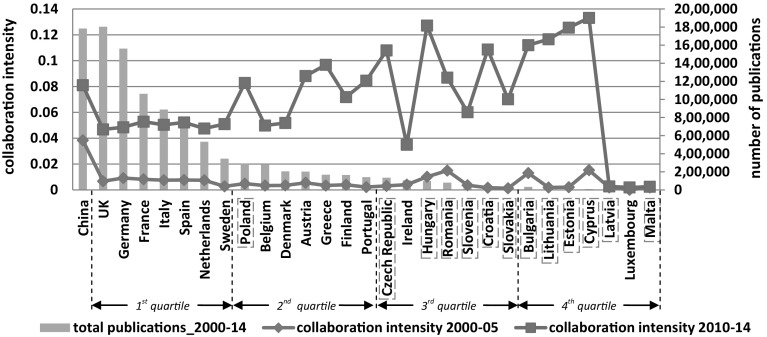
Changes of collaboration intensity. *Note* (1) Collaboration intensity is the average of the collaboration strength index calculated by Eq. (1). (2) Four quartiles are divided based on the number of publications

To reflect the general level of international collaboration strength between one country and the other 28 partners, the mean was taken after calculating the Jaccard collaboration strength (Eq. 1) in the matrix. Figure 1 displays the averaged collaboration strength (which is called collaboration intensity in the figure) as well as the scientific capacity of each country (proxied by the total publications during the period of 2000 and 2014).^11^

During the studied 15 years, the UK and Germany published over 1.5 million articles, which was similar to the amount of publications by Chinese researchers. In the EU28, the old members, such as the UK, Germany, France, Italy, Spain, the Netherlands and Sweden, were in the top quartile, representing the scientifically strong countries. Located in the second and third quartile, countries between Poland and Slovakia can be defined as the middle-level-capacity countries. Latvia, Luxembourg and Malta had the fewest publications during the studied 15 years.

Figure 1 depicts the collaboration propensity (i.e. intensity of collaborations between China and the EU28, measured by the Jaccard index) and research capacity (i.e. number of total publications at national level). We label the 13 new EU members in light blue squares. To offer a dynamic view, the collaboration intensity is provided for two time periods, i.e. the earlier stage between 2000 and 2005 (blue line) and the later stage between 2010 and 2014 (red line).

For China, the intensity of collaborations with the whole EU has increased from 0.04 to 0.08. In European countries, as shown in Fig. 1, the research capacity and collaboration intensity are considerably heterogeneous. The EU countries with strong research capacity (proxied by the number of total publications at national level) are the old EU members, such as UK, Germany, France, Italy, Spain, the Netherlands and Sweden. In these countries, the intensity of collaborations with China increased almost equally, from on average 0.7 to 5%. However, countries in the 2nd, 3rd and part of the 4th quartile, which represent middle- and low-level-capacity countries, have increased their collaboration intensity most. Austria, Greece and Portugal are the three old EU members who have advanced their collaboration strength remarkably, to above 8% by the later stage. Poland is the only new EU member state which is located in the 2nd quartile in terms of scientific capacity.^12^ As an old EU member, Ireland’s scientific capacity is positioned in the third quartile, and its collaboration intensity stays relatively low in the second stage. All the new member states, except Latvia and Malta, have improved their collaboration intensity to around 10% or even more. Being an old EU member, Luxembourg’s Jaccard index remained low in both stages. This indicates that, together with Latvia and Malta, Luxembourg was isolated in the collaboration network with China.

### The change of centrality of the collaboration network

In the process of collaborating with China, due to the geographical location and historical factors, etc., European countries tend to have preferential partners within the EU. Betweenness centrality is an important structural attribute in co-publication networks, and nodes with a high centrality degree play the roles of being brokers or gatekeepers to connect the nodes and sub-groups (Freeman 1978; Abbasi et al. 2012). The betweenness centrality helps to understand the position of a country in the knowledge flow. A higher centrality represents a more important as well as more advantageous position of this country (Leydesdorff and Wagner 2008). The EU countries with a high level of centrality are regarded as the important nodes in connecting China and other EU member states.

**Fig. 2:**
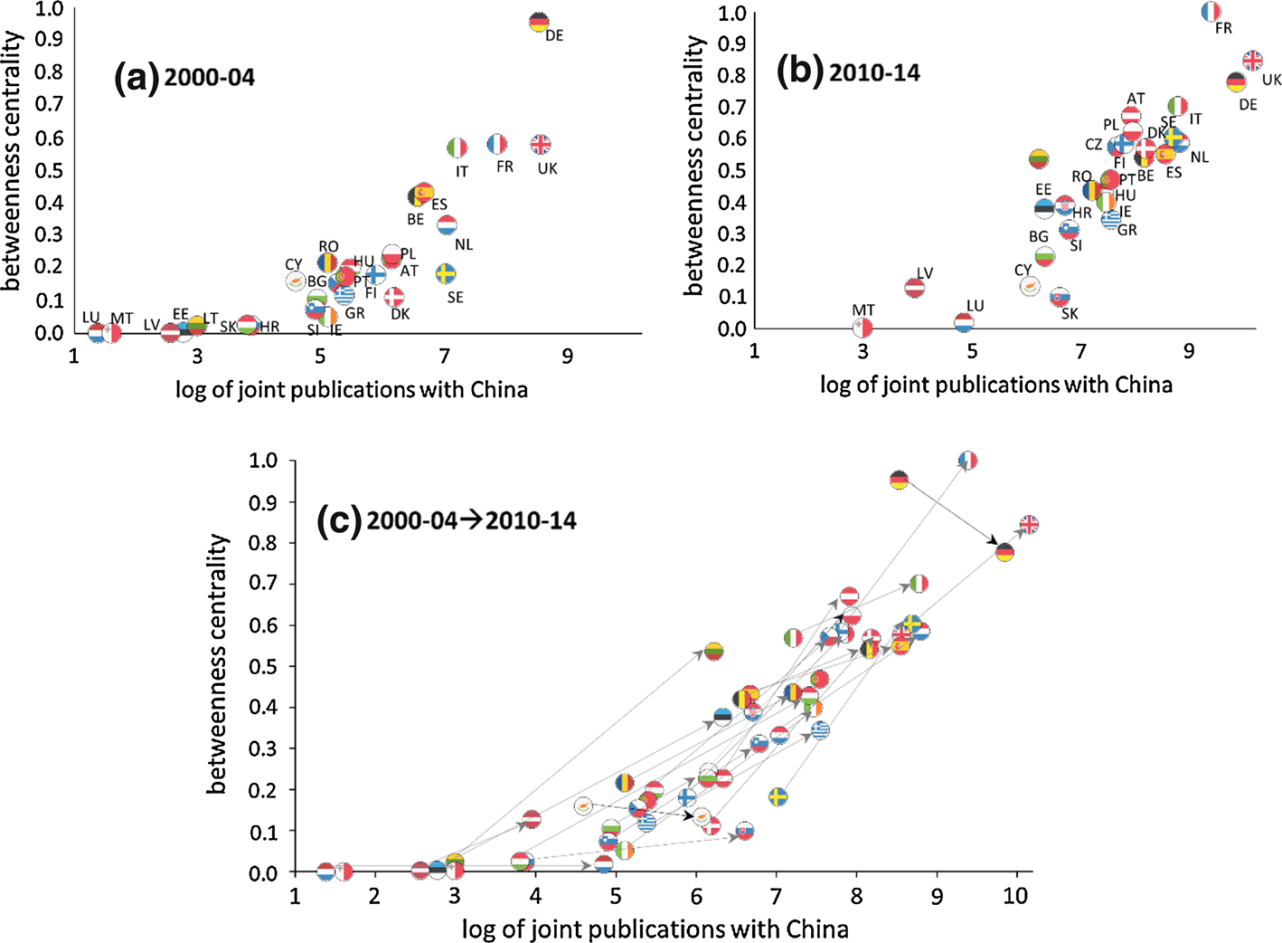
Shift of network centrality in the China-EU28 collaboration network. *Note* The values plotted are normalized betweenness centrality (see Eq. 3). *Source* Authors’ own elaboration

Figure 2 provides the degree changes of the 28 European countries over the years. In Fig. 2, the *y* axis represents the degree of betweenness centrality and the x-axis represents the logarithm value of the number of articles jointly published with China.

In the earlier stage 2000–2004, illustrated in Fig. 2a, the superpower Germany dominated the central position, with the highest degree of betweenness centrality. Following that, other high-capacity countries like the UK, France, Italy, Spain and the Netherlands also had a relatively high level of betweenness centrality. In the later stage 2010–2014, presented in Fig. 2b, the betweenness centrality of the UK and France had increased tremendously. There was, however, a dramatic decline for the betweenness centrality of Germany. In fact, Germany was one of the only two countries with the centrality decreasing, while the other country was Cyprus. As shown in Fig. 2b, in the later stage, France, the UK, and Germany had similar levels of betweenness centrality. Figure 2c captures the changes of countries between these two stages. From the earlier stage to the later stage, almost all the countries have increased both the volume of collaborated publications and the betweenness centrality, which can be reflected by the moving trend from the lower left to the upper right in the scatter plot in Fig. 2c.

### Evolution of collaboration networks

In order to understand the pattern of knowledge exchange in the process of China-EU28 scientific collaborations, this section maps the collaboration network and detects its changes. Special attention will be paid to the change of collaboration patterns of new member states (after they joined the EU), as well as the interactions between the new and old member states.

**Fig. 3:**
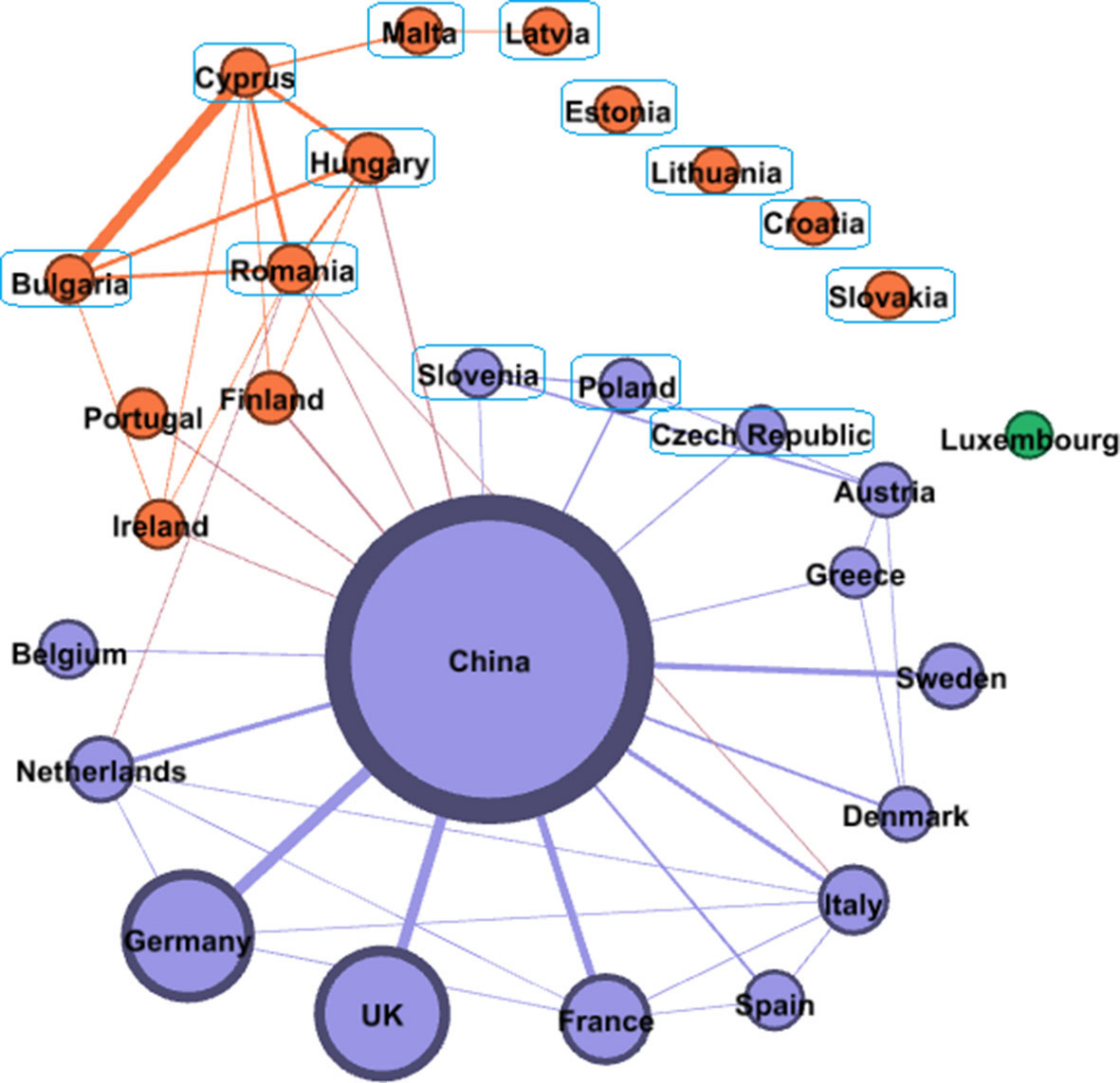
The structure of China-EU28 collaboration network (2000–2004). *Note* (1) The size of the *circle* represents the volume of joint publications with China between 2000 and 2004. (2) The thickness of the *lines* represents the connection strength between countries. (3) Network filter is used in order to have a clear map. (4) Different node colours indicate different clusters. (5) Thirteen new EU members are in *light blue squares*. *Source* Authors’ own elaboration. (Color figure online)

**Fig. 4:**
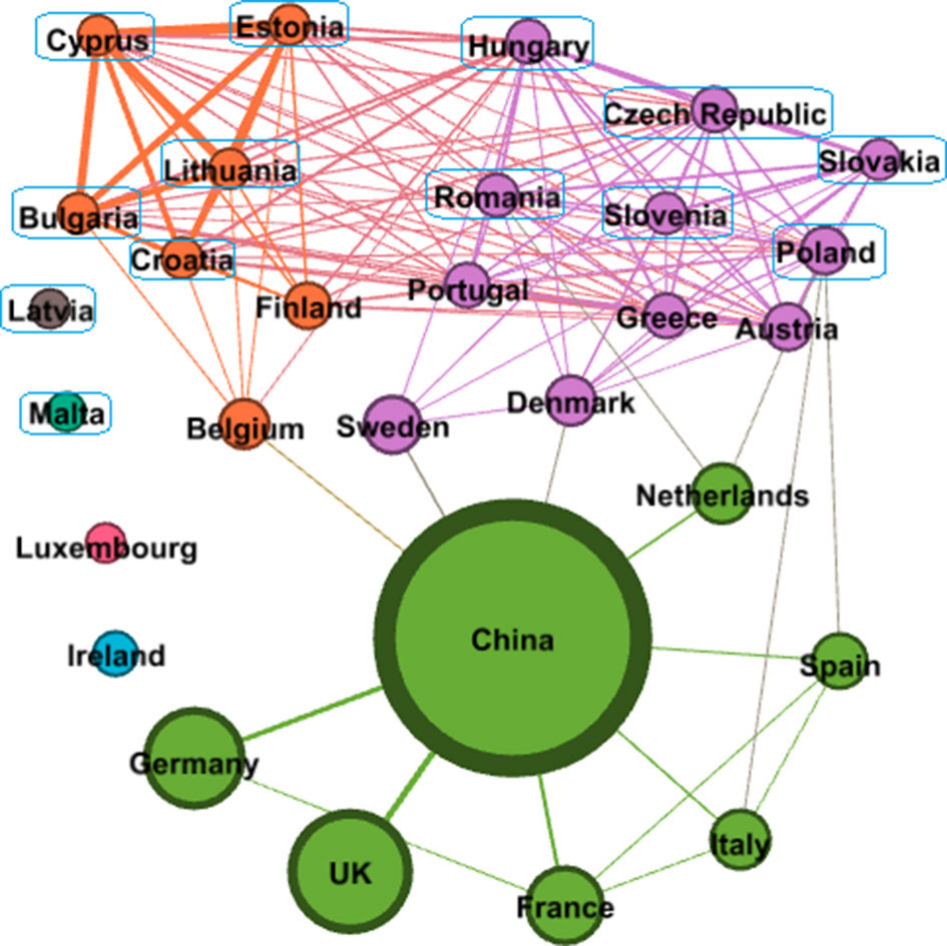
The structure of China-EU28 collaboration network (2010–2014). *Note* (1) The size of the *circle* represents the volume of joint publications with China between 2010 and 2014. (2) The thickness of the *lines* represents the connection strength between countries. (3) Network filter is used in order to have a clear map. (4) Different node colours indicate different clusters. (5) Thirteen new EU members are in *light blue squares*. *Source* Authors’ own elaboration. (Color figure online)

Figures 3 and 4 provide the collaboration network structure in two different stages, an earlier stage of 2000–2004 and a later stage of 2010–2014. Publications covered in the earlier stage are related to research activities before the thirteen member states joined the EU.^13^ In the network figures, new EU members are labelled in light blue squares.

As shown in Fig. 3, there are two main clusters in the earlier stage. Led by the high research capacity countries, e.g. Germany, the UK, France, Spain, the Netherlands, etc., a cluster consists of 14 countries plotted at the lower level of the figure. This cluster consists of mainly pre-existing EU members. Three countries—Slovenia, Poland and Czech Republic, who joined the EU in 2004, are also in this cluster. On the top of the figure, there is another cluster mainly formed by new member states. Three old EU members—Portugal, Finland and Ireland—also belong to this group. In this cluster, Cyprus, Bulgaria, Hungary and Romania in particular are strongly connected with each other. Though belonging to the same cluster, Slovakia, Croatia, Lithuania and Estonia are hardly connected with others.^14^ Luxembourg is isolated from either of the main clusters.

At the later stage (Fig. 4), after the 13 new members have also joined the EU, the main clusters have developed from two to three. The connections between the high capacity old EU members (i.e. Germany, the UK, France, Italy, Spain, The Netherlands) are sparse. Interestingly, however, many of the new EU member states have tremendously increased their collaboration links with each other.

Another interesting observation from Fig. 4 is that some old EU member states (i.e. Belgium, Sweden, Denmark and the Netherlands) are located in very important positions in the network. It has been shown in “The change of centrality of the collaboration network” section (Fig. 2) that these countries have increased their betweenness centrality remarkably in the later stage. If these nodes are removed from the recent network, the majority of the new EU members can hardly be connected with China any more. In other words, these old EU member states act as the brokers linking up China and the new EU member states. Hence, knowledge exchange between China and these new EU member states—that are middle and low capacity countries—crucially depend on the brokers who are connecting the network.

Given that all the publications from our dataset are those jointly published with China, the figures reveal that the small countries (on the top of the figures) collaborate with China together with the brokers, e.g. Belgium, Sweden, Denmark and the Netherlands. The figures also illustrate that there is a very strong clustering effect on the top of Fig. 4, which consists of countries with middle- or low- research capacity,^15^ including mostly new EU member states and a few old member states. On the top left, Finland and Belgium belong to the cluster which includes the new members such as Bulgaria, Cyprus, Estonia, Lithuania and Croatia. On the right top, the cooperation cluster includes five old EU members (Sweden, Denmark, Austria, Greece and Portugal) and six new EU members (Hungary, Czech Republic, Slovenia, Slovakia, Romania and Poland).

Comparing the two networks (Figs. 3, 4), judging from the link strength between countries, it shows that the knowledge integration in the new EU member states have increased remarkably. This signals that joining the EU has exerted an obvious influence on scientific integrations between these new member states. However, there are still some countries, e.g. Luxembourg and Ireland, which are relatively isolated. To facilitate a strong integration, special attention needs to be paid to the isolated countries.

Middle-sized countries are the most dynamic players in the process of network evolution. While the superpowers like Germany and the United Kingdom collaborate with China directly without other European countries being involved, the new EU member states—that have a relatively low scientific capacity—intend to cooperate with China jointly with old EU member(s). The dense connections between the new EU members (with middle- or low-capacity) reveal the emergence of research economies in Europe.

## Conclusions

In the globalization era, scientific collaborations between China and the EU member states have been greatly strengthened. To further facilitate and benefit from knowledge flows, it is of importance to understand the structure and dynamics of collaboration networks. What is more important for the EU side, the intra-European collaboration appears to be embedded within the network of collaborations with China. Based on co-authorship data between 2000 and 2014 this paper examines the network structure of collaborations between China and the EU 28, as well as the interactions between EU member states.

Our results show that the new China-EU collaboration network has been reshaped in particular by a number of new EU member states that have middle or low research capacities. These emerging research economies, as indicated by Adams (2012), are changing the global balance of research activity. There are several reasons behind this phenomenon. On one hand, collaborations between China and ‘old’ EU member states—such as Germany, the UK and France—have been established for a relatively long time, and the international partnership stays rather stable. However, there is not much increasing space as the scientists in such large countries have a relatively higher possibility to collaborate with domestic partners (Frenken 2002). On the other hand, there is much potential for collaboration between China and the middle- and low capacity nations as their research is more internationally oriented. More and more Chinese researchers have come to realize that there are also many excellent institutes and researchers besides those in the large countries and have begun to seek collaborators from a wider range of regions, including those relatively small countries. If the network cohesion represents a positive relationship with the extent of information exchange (Fritsch and Kauffeld-Monz 2010), the majority of the new EU member states have been embedded in a better network structure after they joined the EU.

What’s more, this study provides interesting evidence on the dynamic change of collaborations between the old and new EU member states. In the later years, after joining the EU, the new EU members have presented intensive collaborations with some pre-existing EU members as well as strong interactions among the new members themselves. This phenomenon can be partly explained by the argument of Hoekman et al. (2013), that the EU funding has a significant effect on scientific co-publications between member states that did not intensively co-publish before, and in particular when involving scientifically weak regions. This is also one of the goals of the European Research Area (ERA) concept, which is to increase collaborations between European regions and contribute to geographically integrated European research systems (Scherngell and Lata 2013).

Using a dataset of co-publications between European regions in the period 2000–2007, Hoekman et al. (2010) find a gradual convergence toward a more integrated European science system. Frenken (2002) also points out that small and peripheral countries have been more and more involved in European collaboration. Our results show that, in the process of collaborating with China, such “Europeanisation” phenomenon is even more obvious in recent years (i.e. 2010–2014) after the participation of the new EU member states. Small countries that joined the EU after 2000 have in particular enormously improved their interconnection with other member states.

Another interesting observation from this study is that a few old EU member states play a crucial role in forming the collaboration network. Maes and Verdun (2005) found that, in addition to large EU member states, small countries such as Belgium and the Netherland have played a significant role in the EU, e.g. in the creation of the economic and monetary union in Europe. Our results demonstrate that, in terms of producing scientific output, countries such as Belgium, Sweden, Denmark and the Netherlands, act as important brokers connecting China and the new EU members.

Evidence shows that the international collaboration propensity in small and peripheral European countries has increased enormously. Following this trend and with the rise of China as a science powerhouse, it is expected that there will be more and more collaborations between China and small European countries. Although having no advanced research resources to provide, such small and peripheral countries can provide complementarities to their partners. Nevertheless, economic distance (in terms of R&D capability) is often a factor impeding core-peripheral collaborations (Acosta et al. 2011; Autant-bernard et al. 2007). In such a case, the middle-level partners, who are open to and in favor of European integration (Maes and Verdun 2005), serve as gatekeepers that play an important role in bridging China and the small partners in the EU.

One limitation of this study is that the networks are mapped based on full count, using one type of collaboration measure (i.e. the Jaccard index). Although the Jaccard index is a commonly used method in analysing co-author networks (Pislyakov and Shukshina 2014; Leydesdorff 2008), one should bear in mind that there are also other indicators, including fractional values, Salton’s indexes and probabilistic measures (Probabilistic Affinity Index and Probabilistic Partnership Index). Comparison between the results employing the probabilistic measures and the Jaccard index is worthy of additional investigation.

## Footnotes

1 We use the terminology ‘old’ and ‘new’ EU Member States to refer to, respectively, the countries that formed the EU15 and those that joined the EU in 2004 or later. For further details, see "Dynamics of scientific collaborations" section.

2 See more at http://eeas.europa.eu/archives/delegations/china/eu_china/research_innovation/st_relations/index_en.htm.

3 See also at http://ec.europa.eu/research/iscp/index.cfm?pg=china.

4 The official document is available at https://eeas.europa.eu/sites/eeas/files/s_t_partnership_agreement_cestys_en.pdf.

5 Based on data from Web of Science.

6 The official guideline of the ‘‘Medium- ‘Medium- and Long-Term Programme for Science and Technology Development’’ (in Chinese) is available on the website of MSTC (Ministry of Science and Technology of the People’s Republic of China) http://www.most.gov.cn/kjgh/kjghzcq/.

7 Salton index is calculated by $\text{CSI} = \text{CO}_{ij} /\sqrt {P_{i} *P_{j} }$.

8 They are Belgium, Germany, France, Italy, Luxembourg and the Netherlands.

9 For a detailed overview of EU history and integration, see e.g.(Craig and De Búrca 2015, pp. 1–23).

10 This includes Austria, Belgium, Denmark, Finland, France, Germany, Greece, Italy, Ireland, Luxembourg, Netherlands, Portugal, Spain, Sweden and UK.

11 Scientific capacity can be evaluated by different indicators, such as total R&D input, R&D per capita or various types of scientific output. Here we use the volume of academic publications to represent the level of scientific capacity.

12 Note again that this is measured only by the volume of total publications.

13 Although 10 countries (Czech Republic, Estonia, Cyprus, Latvia, Lithuania, Hungary, Malta, Poland, Slovenia and Slovakia) joined the EU in May 2004, the publications indexed in 2004 reflect research activities that were carried out some time before the publication date, i.e. before they joined the EU.

14 In order to show the main linkages, network filter has been applied. Thus the weak edges are not invisible in the network figures.

15 Research capacity is proxied by the number of publications (see more in "Dynamics of scientific collaborations" section, Fig. 1).
